# Supplementary material for: Genetic Screening Reveals Heterogeneous Clinical Phenotypes in Patients with Dilated Cardiomyopathy and Troponin T2 Variants
Source: J Pers Med. 2023 Mar 31;13(4):611. doi: 10.3390/jpm13040611 (PMC10145473; doi:10.3390/jpm13040611)

Supplementary Table S1

Clinvar ID	Gene Symbol	Variant Type	Consequence	ClinicalSignificance	Status	Reported phenotypes	Variant identifier
12408	*TNNT2*	SNV	Missense	Pathogenic	Criteria provided/ multiple submitters/ no conflicts	Hypertrophic cardiomyopathy<br>Left ventricular noncompaction<br>Restrictive cardiomyopathy	NM_001276345.2(TNNT2): c.266T>A p.(Ile89Asn)
12409	*TNNT2*	SNV	Missense	Pathogenic/Likely pathogenic	Criteria provided/ multiple submitters/ no conflicts	Hypertrophic cardiomyopathy<br>restrictive cardiomyopathy<br>Left ventricular noncompaction	NM_001276345.2(TNNT2): c.305G>A p.(Arg102Gln)
12412	*TNNT2*	SNV	Missense	Pathogenic	Criteria provided/ single submitter	Familial hypertrophic cardiomyopathy<br>Left ventricular noncompaction	NM_001276345.2(TNNT2): c.358T>A p.(Phe120Ile)
12414	*TNNT2*	SNV	Missense	Pathogenic	Criteria provided/ multiple submitters/ no conflicts	Left ventricular noncompaction<br>Dilated cardiomyopathy<br>Hypertrophic cardiomyopathy<br>Familial restrictive cardiomyopathy	NM_001276345.2(TNNT2): c.451C>T p.(Arg151Trp)
12415	*TNNT2*	SNV	Missense	Pathogenic/Likely pathogenic	Criteria provided/ multiple submitters/ no conflicts	Left ventricular noncompaction<br>Dilated cardiomyopathy<br>Familial restrictive<br>Hypertrophic cardiomyopathy	NM_001276345.2(TNNT2): c.421C>T p.(Arg141Trp)
12416	*TNNT2*	SNV	Missense	Pathogenic	No assertion criteria provided	Left ventricular noncompaction	NM_001276345.2(TNNT2): c.644G>T p.(Arg215Leu)
12417	*TNNT2*	SNV	Missense	Pathogenic	No assertion criteria provided	Left ventricular noncompaction	NM_001276345.2(TNNT2): c.838G>A p.(Asp280Asn)
43625	*TNNT2*	SNV	Missense	Likely pathogenic	Criteria provided/ single submitter	Dilated cardiomyopathy	NM_001276345.2(TNNT2): c.294T>G p.(Asp98Glu)
43626	*TNNT2*	SNV	Missense	Likely pathogenic	Criteria provided/ multiple submitters/ no conflicts	Hypertrophic cardiomyopathy<br>Left ventricular noncompaction<br>Familial restrictive cardiomyopathy	NM_001276345.2(TNNT2): c.287A>C p.(Asp96Ala)
43627	*TNNT2*	SNV	Missense	Pathogenic	Criteria provided/ multiple submitters/ no conflicts	Hypertrophic cardiomyopathy<br>Left ventricular noncompaction<br>Restrictive cardiomyopathy	NM_001276345.2(TNNT2): c.304C>T p.(Arg102Trp)
43628	*TNNT2*	SNV	Missense	Pathogenic	Criteria provided/ multiple submitters/ no conflicts	Hypertrophic cardiomyopathy<br>Left ventricular noncompaction<br>Familial restrictive cardiomyopathy	NM_001276345.2(TNNT2): c.311G>A p.(Arg104His)
43629	*TNNT2*	SNV	Missense	Pathogenic/Likely pathogenic	Criteria provided/ multiple submitters/ no conflicts	Hypertrophic cardiomyopathy<br>Left ventricular noncompaction<br>Restrictive cardiomyopathy	NM_001276345.2(TNNT2): c.311G>T p.(Arg104Leu)
43634	*TNNT2*	SNV	Missense	Likely pathogenic	Criteria provided/ single submitter	Dilated cardiomyopathy	NM_001276345.2(TNNT2): c.382G>A p.(Glu128Lys)
43636	*TNNT2*	SNV	Missense	Pathogenic/Likely pathogenic	Criteria provided/ multiple submitters/ no conflicts	Hypertrophic cardiomyopathy<br>Left ventricular noncompaction<br>Restrictive cardiomyopathy	NM_001276345.2(TNNT2): c.418C>T p.(Arg140Cys)
43637	*TNNT2*	SNV	Missense	Pathogenic	Criteria provided/ multiple submitters/ no conflicts	Dilated cardiomyopathy| Hypertrophic cardiomyopathy<br>Left ventricular noncompaction<br>Restrictive cardiomyopathy	NM_001276345.2(TNNT2): c.422G>A p.(Arg141Gln)
43639	*TNNT2*	SNV	Missense	Likely pathogenic	Criteria provided/ single submitter	Dilated cardiomyopathy	NM_001276345.2(TNNT2): c.430C>G p.(Arg144Gly)

43649	*TNNT2*	SNV	Missense	Pathogenic/Likely pathogenic	Criteria provided/ multiple submitters/ no conflicts	Dilated cardiomyopathy<br>Hypertrophic cardiomyopathy<br>Left ventricular noncompaction<br>Restrictive cardiomyopathy	NM_001276345.2(TNNT2): c.548G>A p.(Arg183Gln)
43673	*TNNT2*	SNV	Splice-D/A	Pathogenic	Criteria provided/ multiple submitters/ no conflicts	Hypertrophic cardiomyopathy<br>Left ventricular noncompaction<br>Restrictive cardiomyopathy	NM_001276345.2(TNNT2): c.851+1G>A
177636	*TNNT2*	SNV	Stop gain	Pathogenic/Likely pathogenic	Criteria provided/ multiple submitters/ no conflicts	Hypertrophic cardiomyopathy<br>Left ventricular noncompaction<br>Restrictive cardiomyopathy	NM_001276345.2(TNNT2): c.890G>A p.(Trp297Ter)
177855	*TNNT2*	SNV	Missense	Likely pathogenic	No assertion criteria provided	Hypertrophic cardiomyopathy	NM_001276345.2(TNNT2): c.837C>A p.(Asn279Lys)
177644	*TNNT2*	SNV	Missense	Pathogenic/Likely pathogenic	Criteria provided/ multiple submitters/ no conflicts	Hypertrophic cardiomyopathy	NM_001276345.2(TNNT2): c.274G>A p.(Gly92Arg)
165533	*TNNT2*	SNV	Splice-D/A	Likely pathogenic	Criteria provided/ single submitter	Hypertrophic cardiomyopathy	NM_001276345.2(TNNT2): c.851+1G>T
177634	*TNNT2*	SNV	Missense	Pathogenic/Likely pathogenic	Criteria provided/ multiple submitters/ no conflicts	Hypertrophic cardiomyopathy<br>Left ventricular noncompaction<br>Restrictive cardiomyopathy	NM_001276345.2(TNNT2): c.566C>T p.(Ser189Phe)
177807	*TNNT2*	SNV	Missense	Pathogenic/Likely pathogenic	Criteria provided/ multiple submitters/ no conflicts	Hypertrophic cardiomyopathy	NM_001276345.2(TNNT2): c.360T>G p.(Phe120Leu)
165549	*TNNT2*	SNV	Missense	Pathogenic/Likely pathogenic	Criteria provided/ multiple submitters/ no conflicts	Restrictive cardiomyopathy<br>Hypertrophic cardiomyopathy<br>Left ventricular noncompaction	NM_001276345.2(TNNT2): c.310C>T p.(Arg104Cys)
181636	*TNNT2*	SNV	Stop gain	Pathogenic/Likely pathogenic	Criteria provided/ multiple submitters/ no conflicts	Hypertrophic cardiomyopathy<br>Restrictive cardiomyopathy<br>Left ventricular noncompaction	NM_001276345.2(TNNT2): c.891G>A p.(Trp297Ter)
181649	*TNNT2*	SNV	Splice-D/A	Pathogenic/Likely pathogenic	Criteria provided/ single submitter	Hypertrophic cardiomyopathy<br>Left ventricular noncompaction<br>Restrictive cardiomyopathy	NM_001276345.2(TNNT2): c.851+1G>C
181628	*TNNT2*	SNV	Missense	Likely pathogenic	Criteria provided/ single submitter	Not provided/ not specified	NM_001276345.2(TNNT2): c.767A>G p.(Glu256Gly)
181625	*TNNT2*	SNV	Missense	Pathogenic	Criteria provided/ single submitter	Hypertrophic cardiomyopathy<br>Left ventricular noncompaction<br>Restrictive cardiomyopathy	NM_001276345.2(TNNT2): c.616C>T p.(Arg206Trp)
181622	*TNNT2*	SNV	Missense	Likely pathogenic	Criteria provided/ single submitter	Not provided/ not specified	NM_001276345.2(TNNT2): c.526A>G p.(Arg176Gly)
181616	*TNNT2*	SNV	Missense	Likely pathogenic	Criteria provided/ single submitter	Not provided/ not specified	NM_001276345.2(TNNT2): c.412G>A p.(Glu138Lys)
181615	*TNNT2*	SNV	Stop gain	Pathogenic	Criteria provided/ single submitter	Not provided/ not specified	NM_001276345.2(TNNT2): c.349G>T p.(Glu117Ter)
181642	*TNNT2*	SNV	Missense	Likely pathogenic	Criteria provided/ single submitter	Hypertrophic cardiomyopathy	NM_001276345.2(TNNT2): c.307A>C p.(Lys103Gln)
181641	*TNNT2*	SNV	Missense	Pathogenic	Criteria provided/ single submitter	Not provided/ not specified	NM_001276345.2(TNNT2): c.269C>T p.(Pro90Leu)
181610	*TNNT2*	SNV	Missense	Likely pathogenic	Criteria provided/ single submitter	Not provided/ not specified	NM_001276345.2(TNNT2): c.259C>A p.(Pro87Thr)
217496	*TNNT2*	SNV	Missense	Likely pathogenic	Criteria provided/ single submitter	Hypertrophic cardiomyopathy	NM_001276345.2(TNNT2): c.662T>C p.(Ile221Thr)

224775	*TNNT2*	SNV	Missense	Pathogenic	No assertion criteria provided	Left ventricular noncompaction	NM_001276345.2(TNNT2): c.316G>A p.(Glu106Lys)
228409	*TNNT2*	SNV	Missense	Pathogenic	Criteria provided/ multiple submitters/ no conflicts	Dilated cardiomyopathy| Hypertrophic cardiomyopathy Restrictive cardiomyopathy Left ventricular noncompaction	NM_001276345.2(TNNT2): c.547C>T p.(Arg183Trp)
235064	*TNNT2*	SNV	Missense	Likely pathogenic	No assertion criteria provided	Not provided/ not specified	NM_001276345.2(TNNT2): c.358T>C p.(Phe120Leu)
235063	*TNNT2*	SNV	Missense	Pathogenic	No assertion criteria provided	Not provided/ not specified	NM_001276345.2(TNNT2): c.305G>T p.(Arg102Leu)
389145	*TNNT2*	SNV	Missense	Likely pathogenic	Criteria provided/ single submitter	Not provided/ not specified	NM_001276345.2(TNNT2): c.421C>G p.(Arg141Gly)
426997	*TNNT2*	SNV	Splice-D/A	Likely pathogenic	Criteria provided/ single submitter	Not provided/ not specified	NM_001276345.2(TNNT2): c.610-1G>A
430390	*TNNT2*	SNV	Missense	Likely pathogenic	Criteria provided/ single submitter	Not provided/ not specified	NM_001276345.2(TNNT2): c.445C>A p.(Arg149Ser)
431937	*TNNT2*	SNV	Missense	Pathogenic	Criteria provided/ single submitter	Not provided/ not specified	NM_001276345.2(TNNT2): c.847A>G p.(Lys283Glu)
562477	*TNNT2*	SNV	Splice-D/A	Likely pathogenic	No assertion criteria provided	Not provided/ not specified	NM_001276345.2(TNNT2): c.719+2T>A
567696	*TNNT2*	SNV	Missense	Likely pathogenic	Criteria provided/ single submitter	Hypertrophic cardiomyopathy Left ventricular noncompaction Restrictive cardiomyopathy	NM_001276345.2(TNNT2): c.517G>A p.(Glu173Lys)
666939	*TNNT2*	SNV	Missense	Likely pathogenic	Criteria provided/ single submitter	Dilated cardiomyopathy	NM_001276345.2(TNNT2): c.294T>A p.(Asp98Glu)
684831	*TNNT2*	SNV	Missense	Likely pathogenic	Criteria provided/ single submitter	Dilated cardiomyopathy	NM_001276345.2(TNNT2): c.385G>C p.(Glu129Gln)
691648	*TNNT2*	SNV	Splice-D/A	Likely pathogenic	Criteria provided/ single submitter	Cardiomyopathy	NM_001276345.2(TNNT2): c.163+1G>T
691967	*TNNT2*	SNV	Missense	Pathogenic	Criteria provided/ single submitter	Left ventricular noncompaction	NM_001276345.2(TNNT2): c.548G>T p.(Arg183Leu)
1175037	*TNNT2*	SNV	Stop gain	Pathogenic	No assertion criteria provided	Not provided/ not specified	NM_001276345.2(TNNT2): c.844C>T p.(Gln282Ter)
43648	*TNNT2*	Microsatellite	In frame indel	Pathogenic	Criteria provided/ multiple submitters/ no conflicts	Hypertrophic cardiomyopathy Left ventricular noncompaction Restrictive cardiomyopathy	NM_001276345.2(TNNT2): c.508GAG[3] p.(Glu173del)
43659	*TNNT2*	Microsatellite	In frame indel	Pathogenic/Likely pathogenic	Criteria provided/ multiple submitters/ no conflicts	Left ventricular noncompaction Dilated cardiomyopathy Hypertrophic cardiomyopathy Restrictive cardiomyopathy	NM_001276345.2(TNNT2): c.650AGA[3] p.(Lys220del)
177683	*TNNT2*	Deletion	In frame indel	Likely pathogenic	No assertion criteria provided	Hypertrophic cardiomyopathy	NM_001276345.2(TNNT2): c.316_318del p.(Glu106del)
181639	*TNNT2*	Duplication	Frameshift	Pathogenic	Criteria provided/ single submitter	Cardiomyopathy	NM_001276345.2(TNNT2): c.844dup p.(Gln282fs)
229336	*TNNT2*	Indel	Missense	Likely pathogenic	Criteria provided/ single submitter	Dilated cardiomyopathy	NM_001276345.2(TNNT2): c.354_355delinsGT p.(His119Tyr)
636610	*TNNT2*	Deletion	Intronic	Likely pathogenic	Criteria provided/ single submitter	Not provided/ not specified	NM_001276345.2(TNNT2): c.609+1del
684850	*TNNT2*	Deletion	In frame indel	Pathogenic	Criteria provided/ single submitter	Hypertrophic cardiomyopathy	NM_001276345.2(TNNT2): c.328_333del p.(Asn110_Glu111del)

SNV and SV Variants in Clinvar [1] reported in the context of *TNNT2* (NM_001276345.2) were retrieved (as of March 2023) using the SimpleClinvar tool [2]. Included variants were restricted to entries with clinical significance of pathogenic or likely pathogenic.

References:

[1] Landrum MJ, Lee JM, Benson M, Brown GR, Chao C, Chitipiralla S, Gu B, Hart J, Hoffman D, Jang W, Karapetyan K, Katz K, Liu C, Maddipatla Z, Malheiro A, McDaniel K, Ovetsky M, Riley G, Zhou G, Holmes JB, Kattman BL, Maglott DR. ClinVar: improving access to variant interpretations and supporting evidence. Nucleic Acids Res . 2018 Jan 4. PubMed PMID: 29165669 .

[2] Eduardo Perez-Palma, Marie Gramm, Peter Nürnberg, Patrick May and Dennis Lal. Simple ClinVar: an interactive web server to explore and retrieve gene and disease variants aggregated in ClinVar database ***. Nucleic Acids Research*** (2019) PMID:31114901.

Supplementary Table S2: Classification of the reported variants in accordance to the adapted ACMG classification scheme.

Corresponding family	Reported variant	Variant classification according to ACMG guidelines	ACMG criteria applicable
A	NM_001276345.2(TNNT2):c.316G>A p.(Glu106Lys)	Likely pathogenic	PM1, PS4_mod, PP3_mod, PS3_sup, PM2_sup, PP1
B	NM_001276345.2(TNNT2):c.659_661del p.(Lys220del)	Pathogenic	PS3, PS4, PS1, PM4_sup, PM2_sup, PP3,
C	NM_001276345.2(TNNT2):c.644G>C p.(Arg215Pro)	Likely pathogenic	PM1, PM5, PP3_mod, PM2_sup, PP1

Family A:

The variant was found in a hotspot as shown by the absence of benign variants and the colocalization of multiple pathogenic variants close to the reported variant (PM1). The variant has an extremely low population frequency as shown by its absence from gnomAD (PM2_sup). The in-silico predictor REVEL suggests a deleterious effect (REVEL = 0.91) (PP3_mod). A previous publication has published this variant Luedde et al., referred to as NM_001001430.3:c.286G>A p.(Glu96Lys) in multiple family members with left ventricular non-compaction CM. A generated transgenic mouse model of this variant displayed a DCM phenotype including left ventricular dilatation and decreased contractility (PS3_sup). Considering the variable age and severity of disease-onset, the co-segregation of the variant within family A can be considered at supporting level (PP1_sup). In combination, these criteria result in a classification of the presented variant as likely pathogenic.

Family B:

This variant is also referred to as ΔK210 or ΔK207 based on the underlying reference transcript. It leads to a small deletion at the protein level in TNNT2 (PM4_sup). In-silico predictors suggest a deleterious effect (MutationTaster) (PP3). The variant has an extremely low population frequency as shown by its absence from gnomAD (PM2_sup). Multiple previous studies have shown a functional impact illustrated by a decrease in Ca^{2+} sensitivity and a reduced inter-troponin interaction (e.g. Bollen et al. 2017, Venkatraman et al. 2005, Bai et al. 2013, Mogensen et al. 2004) (PS3). The variant has repeatedly been reported as pathogenic (Clinvar ID 43659) (PS1). Furthermore, affected carriers of this variant have previously been reported (Bollen et al. 2017, Miller et al. 2017, Te Rijdt et al. 2019, Golbus et al. 2014)(PS4). Co-segregation of the variant has been extensively shown in the presented family as well as in previous reports (Golbus et al. 2014)(PP1_str). In combination, these criteria result in a classification of the presented variant as pathogenic.

Family C:

The variant was found in a hotspot as shown by the absence of benign variants and the colocalization of multiple pathogenic variants close to the reported variant (PM1). A co-located, (likely) pathogenic variant is published (e.g. Clinvar-ID 180554) (PM5). The variant has an extremely low population frequency as shown by its absence from gnomAD (PM2_sup). The in-silico predictor REVEL suggests a deleterious effect (REVEL = 0.92) (PP3_mod). The co-segregation of the variant within family C can be considered at supporting level (PP1). In combination, these criteria result in a classification of the presented variant as likely pathogenic.

Supplementary Table S3: Conservation of the reported residues. Amino-acid positions correspond to the human *TNNT2* transcript (NM_001276345.2), * implies conservation of the amino acid.

Residue: / Organism:	106	215	220
Homo sapiens	E	R	K
Gorilla gorilla	*	*	*
Canis familiaris	*	*	*
Rattus norvegicus	*	*	*
Mus musculus	*	*	*
Gallus gallus	*	*	*
Danio rerio	*	*	*
Xenopus tropicalis	*	*	M

General publications for ACMG-classification:

Jarvik GP, Browning BL. Consideration of Cosegregation in the Pathogenicity Classification of Genomic Variants. Am J Hum Genet. 2016 Jun 2;98(6):1077-1081. doi: 10.1016/j.ajhg.2016.04.003. Epub 2016 May 26. PMID: 27236918; PMCID: PMC4908147.

Richards S, Aziz N, Bale S, et al. Standards and guidelines for the interpretation of sequence variants: a joint consensus recommendation of the American College of Medical Genetics and Genomics and the Association for Molecular Pathology. *Genet Med*. 2015;17(5):405-424. PMID: 25741868.

Pejaver V, Byrne AB, Feng BJ, et al. Calibration of computational tools for missense variant pathogenicity classification and ClinGen recommendations for PP3/BP4 criteria. *Am J Hum Genet*. 2022;109(12):2163-2177. PMID: 36413997.

Brnich SE, Abou Tayoun AN, Couch FJ, et al. Recommendations for application of the functional evidence PS3/BS3 criterion using the ACMG/AMP sequence variant interpretation framework. *Genome Med*. 2019;12(1):3. Published 2019 Dec 31. PMID: 31892348.

Ellard et *al,* GS Best Practice Guidelines for Variant Classification in Rare Disease 2020.

Ioannidis NM, Rothstein JH, Pejaver V, et al. REVEL: An Ensemble Method for Predicting the Pathogenicity of Rare Missense Variants. *Am J Hum Genet*. 2016;99(4):877-885. PMID: 27666373.

Schwarz JM, Cooper DN, Schuelke M, Seelow D. MutationTaster2: mutation prediction for the deep-sequencing age. Nat Methods. 2014 Apr;11(4):361-2. doi: 10.1038/nmeth.2890. PMID: 24681721.

Citations for variant description:

Van Driest SL, Ellsworth EG, Ommen SR, Tajik AJ, Gersh BJ, Ackerman MJ. Prevalence and spectrum of thin filament mutations in an outpatient referral population with hypertrophic cardiomyopathy. Circulation. 2003 Jul 29;108(4):445-51. doi: 10.1161/01.CIR.0000080896.52003.DF. Epub 2003 Jul 14. PMID: 12860912.

Coppini R, Ho CY, Ashley E, Day S, Ferrantini C, Girolami F, Tomberli B, Bardi S, Torricelli F, Cecchi F, Mugelli A, Poggesi C, Tardiff J, Olivotto I. Clinical phenotype and outcome of hypertrophic cardiomyopathy associated with thin-filament gene mutations. J Am Coll Cardiol. 2014 Dec 23;64(24):2589-2600. doi: 10.1016/j.jacc.2014.09.059. PMID: 25524337; PMCID: PMC4270453.

Murphy SL, Anderson JH, Kapplinger JD, Kruisselbrink TM, Gersh BJ, Ommen SR, Ackerman MJ, Bos JM. Evaluation of the Mayo Clinic Phenotype-Based Genotype Predictor Score in Patients with Clinically Diagnosed Hypertrophic Cardiomyopathy. J Cardiovasc Transl Res. 2016 Apr;9(2):153-61. doi: 10.1007/s12265-016-9681-5. Epub 2016 Feb 25. PMID: 26914223; PMCID: PMC4907543.

Bollen IAE, Schuldt M, Harakalova M, Vink A, Asselbergs FW, Pinto JR, Krüger M, Kuster DWD, van der Velden J. Genotype-specific pathogenic effects in human dilated cardiomyopathy. J Physiol. 2017 Jul 15;595(14):4677-4693. doi: 10.1113/JP274145. Epub 2017 Jun 1. PMID: 28436080; PMCID: PMC5509872.

Venkatraman G, Gomes AV, Kerrick WG, Potter JD. Characterization of troponin T dilated cardiomyopathy mutations in the fetal troponin isoform. J Biol Chem. 2005 May 6;280(18):17584-92. doi: 10.1074/jbc.M409337200. Epub 2004 Dec 28. PMID: 15623536.

Bai F, Caster HM, Pinto JR, Kawai M. Analysis of the molecular pathogenesis of cardiomyopathy-causing cTnT mutants I79N, ΔE96, and ΔK210. Biophys J. 2013 May 7;104(9):1979-88. doi: 10.1016/j.bpj.2013.04.001. PMID: 23663841; PMCID: PMC3647163.

Mogensen J, Murphy RT, Shaw T, Bahl A, Redwood C, Watkins H, Burke M, Elliott PM, McKenna WJ. Severe disease expression of cardiac troponin C and T mutations in patients with idiopathic dilated cardiomyopathy. J Am Coll Cardiol. 2004 Nov 16;44(10):2033-40. doi: 10.1016/j.jacc.2004.08.027. PMID: 15542288.

Miller EM, Hinton RB, Czosek R, Lorts A, Parrott A, Shikany AR, Ittenbach RF, Ware SM. Genetic Testing in Pediatric Left Ventricular Noncompaction. Circ Cardiovasc Genet. 2017 Dec;10(6):e001735. doi: 10.1161/CIRCGENETICS.117.001735. PMID: 29212898; PMCID: PMC8428628.

Te Rijdt WP, Hoedemaekers YM, Jongbloed JDH, Damman K, van der Zwaag PA, de Boer RA, Maass AH, van den Berg MP. Dyssynchronopathy Can be a Manifestation of Heritable Cardiomyopathy. Circ Genom Precis Med. 2019 May;12(5):e002528. doi: 10.1161/CIRCGEN.119.002528. Erratum in: Circ Genom Precis Med. 2019 Jun;12(6):e000056. PMID: 31112419.

Golbus JR, Puckelwartz MJ, Dellefave-Castillo L, Fahrenbach JP, Nelakuditi V, Pesce LL, Pytel P, McNally EM. Targeted analysis of whole genome sequence data to diagnose genetic cardiomyopathy. Circ Cardiovasc Genet. 2014 Dec;7(6):751-759. doi: 10.1161/CIRCGENETICS.113.000578. Epub 2014 Sep 1. PMID: 25179549; PMCID: PMC4270910.

Supplemental Figure S1. Visualisation of protein altering variants in TNNT2. Dots represent missense or inframe variants from Clinvar (corresponding to supplemental table 1). Labeled dots show the position of the three variants reported in this publication. The protein features were annotated based on P45379 (Uniprot).

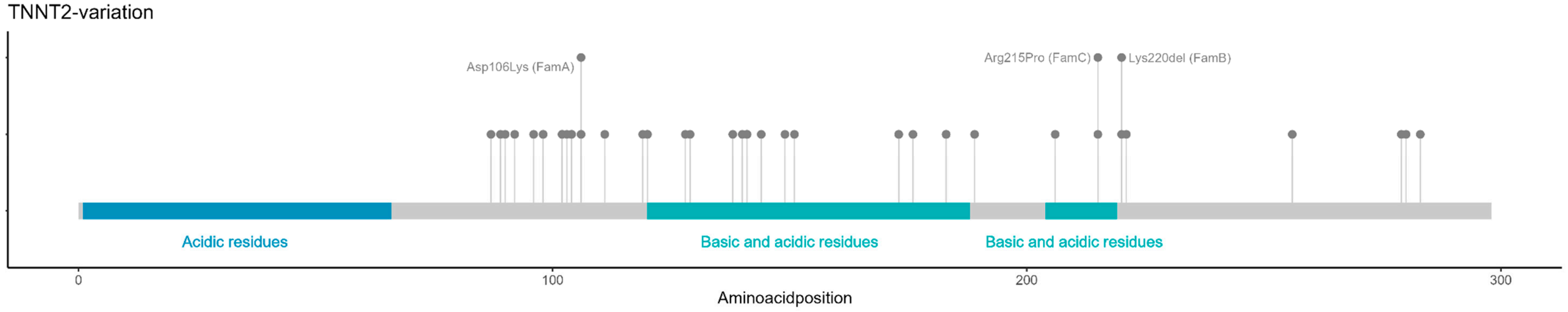

Supplement: Supplementary file 1 [file jpm-13-00611-s001.zip › jpm-2274209-supplementary.pdf]
